# Comparison of pediatric and adult lymphomas involving the mediastinum characterized by distinctive clinicopathological and radiological features

**DOI:** 10.1038/s41598-017-02720-1

**Published:** 2017-05-31

**Authors:** Lei Chen, Mingsong Wang, Hua Fan, Fengqing Hu, Tingting Liu

**Affiliations:** 1 Department of Pathology, Xinhua Hospital, Shanghai Jiaotong University School of Medicine, Shanghai, China; 2 Department of Thoracic Cardiovascular Surgery, Xinhua Hospital, Shanghai Jiaotong University School of Medicine, Shanghai, China; 3 Department of Radiology, Xinhua Hospital, Shanghai Jiaotong University School of Medicine, Shanghai, China; 4 Department of Pediatric Hematology/Oncology, Xinhua Hospital, Shanghai Jiaotong University School of Medicine, Shanghai, China

## Abstract

Lymphomas involving the mediastinum occur in a wide age range and represent heterogeneous histological subtypes with various clinical symptoms and complex radiological findings. However, studies that describe the clinical and radiological features of different subtypes among Chinese pediatric and adult patients are limited. We analyzed the clinical, radiological and pathological features of 31 pediatric lymphomas involving the mediastinum, and compared them to the features of 21 adult patients. Although several histological subtypes were identified in adults, pediatric patients presented with T-cell lymphoblastic lymphoma/T-cell acute lymphoblastic leukemia (T-LBL/T-ALL) and classical Hodgkin lymphomas (CHL) in 24 and 7 cases, respectively. Compared to adults, pediatric patients were more likely to be male (*P* = 0.089) and showed a higher incidence of T-LBL/T-ALL (*P* = 0.001), prevalence of dyspnea (*P* = 0.001), frequency of stage IV tumors (*P* = 0.008), and ratio of tumor diameter to maximum transthoracic diameter (*P* = 0.015). T-LBL/T-ALL patients presented with a higher frequency with stage IV disease (*P* = 0.000 and *P* = 0.001), compression of the blood vessels (*P* = 0.005 and *P* = 0.017), and pleural effusions (*P* = 0.001, for both) than CHL and PMBL patients. Compared to adults, pediatric patients with mediastinal lymphomas presented with exclusive histological subtypes of T-LBL/T-ALL and CHL, which showed distinctive characteristics of histological distribution, clinical presentation and radiological assessments.

## Introduction

Lymphomas account for approximately 12% of total mediastinal tumors and represent heterogeneous histological subtypes^1^. Lymphomas involving the mediastinum exhibit a wide age range, occurring in both pediatric and adult patients^2^.

T-cell lymphoblastic lymphoma (T-LBL)/T-cell acute lymphoblastic leukemia (T-ALL) occurs most frequently in childhood but is also observed in adults^3^. Classical Hodgkin lymphoma (CHL) peaks at 15–35 years of age and in late life, and it usually presents with cervical lymphadenopathy. CHL patients often have mediastinal masses^4^. Primary mediastinal large B-cell lymphoma (PMBL), which arises from thymic medullary B-cells, typically occurs in young adults^5^. Mucosa-associated lymphoid tissue (MALT) lymphoma, diffuse large B cell lymphoma (DLBCL), and mediastinal gray-zone lymphoma have also been reported in adults and elderly individuals^6, 7^.

The clinical and radiological presentations of lymphomas involving the mediastinum are complex and nonsepcific^8, 9^. The initial and differential diagnoses of different histological subtypes are confused by clinicians and radiologists in some cases. Moreover, some subtypes demonstrate similar morphological features, representing serious challenges for pathological diagnosis^7, 10^. The clinical and radiological findings are useful to determine the appropriate staging and management.

Previous studies have focused on a single histological subtype, such as T-LBL/T-ALL or PMBL. However, detailed descriptions of clinicopathological and clinicoradiological comparisons between these subtypes are rare. Moreover, few studies have analyzed the clinicopathological features of different subtypes of lymphomas involving the mediastinum in pediatric patients^11–13^. Studies clarifying the differences in histological distribution, clinical spectrum, and radiological features between pediatric and adult patients are limited.

In this study, we conducted a retrospective analysis of the clinical, radiological, morphological, and immunophenotypical features of lymphomas involving the mediastinum, and we further compared the differences between pediatric and adult patients.

## Results

### Morphological and immunohistochemical findings

T-LBL/T-ALL, CHL, PMBL, DLBCL, and MALT lymphomas were identified in 31, 12, 6, 2, and 1 cases, respectively. The immunohistochemical results are shown in Supplementary Tables 1 and 2.

T-LBL/T-ALL were composed of small to medium-sized lymphoblasts with sparse cytoplasm and irregular nuclei. Tumor cells were commonly positive for TDT (21/26, 80.8%), CD3 (29/31, 93.5%), and CD7 (25/27, 92.6%), and they were variably positive for CD5 (16/24, 66.7%), CD99 (14/19, 73.7%), CD2 (12/18, 66.7%), and CD1a (13/21, 61.9%).

CHLs consisted of 11 cases of nodular sclerosis CHL (NSCHL) and 1 case of lymphocyte-rich CHL. NSCHL morphologically characterized in collagen bands surrounding nodules and Reed-Stenberg cells. CHL tumor cells were positive for PAX5 (10/12, 83.3%), CD30 (12/12, 100%), CD15 (9/12, 75.0%), CD20 (4/12, 33.3%) and CD79a (2/9, 22.2%).

PMBLs showed medium to large cells with round or lobulated nuclei and abundant cytoplasm. PMBLs expressed B-cell markers including PAX5 (5/5, 100%), CD20 (5/6, 83.3%) and CD79a (4/5, 80.0%). PMBL tumor cells were frequently positive for CD30 (3/5, 60.0%) and CD23 (4/5, 80.0%).

Systemic DLBCLs involving the mediastinum demonstrated the same pathological features to nodular DLBCLs. The MALT lymphoma was composed of small lymphocytes and monocytoid cells, which were positive for B-cell markers with low Ki-67 expression.

### Clinical characteristics

The clinical characteristics of the 52 patients are summarized in Table 1. The comparison between pediatric and adult patients is shown in Table 2, and the comparisons among the main histological subtypes are shown in Table 3.

**Table 1:** Clinical presentations of 52 cases with lymphomas involving the mediastinum.

Case no.	Age (y)/Gender	Histological subtype	Symptom				LN involvement	LDH (U/L)	Stage	Response/Follow-up (m)
			Cough	Dyspnea	Chest pain	Others				
**Pediatric patients**										
1	4/F	T-LBL/T-ALL	Y	N	N	N	C	896	III	PD/UT
2	5M	T-LBL/T-ALL	Y	N	Y	N	N	563	IV	CR/UT
3	5/M	T-LBL/T-ALL	Y	Y	N	Fever	C	NA	IV	DOD (6)
4	5/F	T-LBL/T-ALL	Y	Y	N	N	N	766	III	CR/UT
5	6/M	T-LBL/T-ALL	N	Y	Y	Neck pain	C	1102	IV	DOD (4)
6	6/M	T-LBL/T-ALL	Y	N	N	Fever	N	398	IV	PR/UT
7	6/F	T-LBL/T-ALL	N	N	N	Neck enlargement	C and A	326	IV	CR/UT
8	6/M	T-LBL/T-ALL	Y	Y	Y	N	C and A	1882	IV	DOD (5)
9	7/M	T-LBL/T-ALL	Y	Y	N	N	Md	635	III	PR/UT
10	7/M	T-LBL/T-ALL	Y	N	N	Fever	Md	NA	IV	CR/NR (10)
11	7/F	T-LBL/T-ALL	N	N	N	Neck enlargement	C	344	IV	CR/NR (15)
12	8/M	T-LBL/T-ALL	Y	Y	N	N	Md	628	IV	CR/NR (30)
13	9/F	T-LBL/T-ALL	Y	Y	N	N	C	419	IV	CR/UT
14	9/M	T-LBL/T-ALL	Y	N	N	N	C	2289	III	CR/UT
15	11/M	T-LBL/T-ALL	Y	N	N	N	C	271	IV	DOD (7)
16	11/M	T-LBL/T-ALL	Y	N	Y	N	N	795	IV	CR/UT
17	11/M	T-LBL/T-ALL	Y	N	N	Fever	C, Ax and Ab	285	IV	PR/UT
18	12/F	T-LBL/T-ALL	N	Y	Y	SVCS	C, Ax and Ab	292	IV	DOD (8)
19	13/M	T-LBL/T-ALL	Y	Y	N	Fever	C, Ax, and Md	759	IV	PD/UT
20	13/M	T-LBL/T-ALL	N	Y	N	Dysphagia	NA	297	III	CR/UT
21	13/M	T-LBL/T-ALL	Y	N	N	Fever	C and Ab	2403	IV	CR/UT
22	14/M	T-LBL/T-ALL	N	Y	N	N	C	NA	IV	CR/NR (32)
23	18/M	T-LBL/T-ALL	Y	N	N	N	C, Ax and Ab	987	IV	CR/NR (12)
24	18/F	T-LBL/T-ALL	Y	N	Y	Fever	C, Ax and Md	386	IV	DOD (7)
25	4/M	CHL	N	N	N	Fever	Abdominal LV	368	III	CR/NR (14)
26	5/M	CHL	Y	N	N	N	C, Ax and Md	378	II	CR/NR (24)
27	5/F	CHL	N	N	N	N	C	252	III	PR/NR (14)
28	7/M	CHL	Y	N	N	Fever	C	NA	II	CR/NR (31)
29	10/F	CHL	Y	N	N	Fever	C and Md	NA	II	CR/NR (32)
30	13/M	CHL	N	Y	Y	N	C and Md	394	III	PR/UT
31	14/M	CHL	N	N	N	Neck enlargement	C, Ax and Md	200	II	CR/UT
**Adult patients**										
32	20/M	T-LBL/T-ALL	Y	N	Y	Fever	C	227	IV	CR/NR (10)
33	20/M	T-LBL/T-ALL	N	N	N	N	C and Md	264	IV	PD/UT
34	28/M	T-LBL/T-ALL	Y	N	N	N	C, Ax, and Ab	2286	IV	CR/UT
35	31/M	T-LBL/T-ALL	Y	N	N	Fever, neck enlargement	C, Ax, Md and Ab	189	IV	PR/NR (32)
36	34/M	T-LBL/T-ALL	Y	N	N	N	C	972	II	PD/UT
37	42/M	T-LBL/T-ALL	N	N	Y	Neck enlargement	C, Md and I	245	III	CR/UT
38	54/F	T-LBL/T-ALL	N	N	Y	Fever	C, Md and I	233	III	PD/Relapsed
39	21/F	CHL	Y	N	N	Fever	C, M	432	III	CR/NR (16)
40	22/F	CHL	N	N	N	N	C	NA	II	CR/NR (35)
41	23/M	CHL	N	N	N	N	C and Md	152	II	CR/NR (20)
42	25/F	CHL	N	N	N	N	C and M	461	III	PR/NR (20)
43	34/F	CHL	N	N	N	N	C	NA	II	CR/NR (14)
44	23/F	PMBL	N	N	Y	N	C	225	III	CR/NR (33)
45	25/F	PMBL	Y	N	N	Neck enlargement	C and Md	156	II	PD/UT
46	27/F	PMBL	N	N	Y	N	NA	NA	II	CR/NR (31)
47	28/F	PMBL	N	N	Y	N	N	NA	I	CR/NR (24)
48	36/M	PMBL	Y	N	N	N	C and Md	NA	II	CR/UT
49	44/F	PMBL	N	N	Y	N	N	NA	I	CR/NR (30)
50	52/F	MALT	N	N	Y	Fever	C and Md	159	II	PR/NR (23)
51	34/M	DLBCL	Y	N	N	Fever	C	356	IV	PR/NR (29)
52	64/M	DLBCL	Y	N	N	N	Md	NA	II	PR/NR (20)

Abbreviations: Ab, abdominal; Ax, axillary; C, cervical; CHL, classical Hodgkin lymphoma; CR, complete remission; D, diffuse large B-cell lymphoma; DOD, dead of disease; F, female; LN, lymph node; I,inguinal; MALT, mucosa associated lymphoid tissue lymphoma; Md, mediastinal node; M, male; N, no; NA, not available; NSCHL, nodular sclerosis classical Hodgkin lymphoma; NR, no relapse; PMBL, primary mediastinal large B-cell lymphoma; PD, progressing disease; PR, partial remission; T-LBL, T-cell lymphoblastic lymphoma/T-cell lymphoblastic leukemia; UT, under treatment; Y, yes; SVCS, superior vena cava syndrome.

**Table 2:** Comparisons of histological subtypes, clinical features, and radiological findings between pediatric and adult patients.

	T, n = 52, no. (%)	P, n = 31, no. (%)	A, n = 21, no. (%)	*P*-value (P vs. A)
Median age, years (range)	13.0 (4–64)	8.0 (4–18)	28.0 (20–64)	
M:F	32:20	22:9	10:11	0.089
**Histological subtype**				
T-LBL/T-ALL	31/52 (59.6)	24/31 (77.4)	7/21 (33.3)	**0.001**
CHL	12/52 (23.1)	7/31 (22.6)	5/21 (23.8)	0.918
PMBL	6/52 (11.5)	0/31 (0)	6/21 (28.6)	**0.003**
MALT	1/52 (1.9)	0/31 (0)	1/21 (4.8)	0.404
DLBCL	2/52 (3.8)	0/31 (0)	2/21 (9.5)	0.158
**Clinical symptom**				
Cough	30/52 (57.7)	21/31 (67.7)	9/21 (42.9)	0.075
Dyspnea	12/52 (23.1)	12/31 (38.7)	0/21 (0)	**0.001**
Chest pain	15/52 (28.8)	7/31 (22.6)	8/21 (38.1)	0.226
Fever	16/52 (30.8)	10/31 (32.2)	6/21 (28.6)	0.777
**Clinical stage**				
Stage I	2/52 (3.8)	0/31 (0)	2/21 (9.5)	0.158
Stage II	13/52 (25.0)	4/31 (12.9)	9/21 (42.9)	**0.034**
Stage III	13/52 (25.0)	8/31 (25.8)	5/21 (23.8)	0.870
Stage IV	24/52 (46.2)	19/31 (61.3)	5/21 (23.8)	**0.008**
Mortality rate	6/52 (11.5)	6/31 (19.4)	0/21 (0)	**0.070**
Tumor size (cm)	9.37 ± 3.24	9.52 ± 3.22	9.07 ± 3.42	0.700
Ratio of TD to MTD	0.47 ± 0.17	0.52 ± 0.17	0.38 ± 0.14	**0.015**
**Tumor compression or encasement**				
Blood vessels encasement	29/35 (82.9)	21/23 (91.3)	8/12 (66.7)	0.151
Pericardiaum	23/35 (65.7)	16/23 (69.6)	7/12 (58.3)	0.709
Trachea	19/35 (54.3)	13/23 (56.5)	6/12 (50.0)	0.736
**Complication**				
Pleural effussion	23/35 (65.7)	18/23 (78.3)	5/12 (41.7)	0.059
Pericardial effusion	14/35 (40.0)	11/23 (47.8)	3/12 (25.0)	0.282
Pneumonia	17/35 (48.6)	11/23 (47.8)	6/12 (25.0)	1.000

Abbreviations: T, total; P, pediatric patients; A, adult patients; TD, tumor diameter; MTD, maximum transthoracic diameter.

**Table 3:** Comparisons of clinical features, and radiographic findings among different histological subtypes.

	Total, n = 52, no. (%)	T-LBL/T-ALL, n = 31, no. (%)	CHL, n = 12, No. (%)	PMBL, n = 6, No. (%)	DLBCL, n = 2, no. (%)	MALT, n = 1, no. (%)	*P*-value (T-LBL/T-ALL vs. CHL)	*P*-value (T-LBL/T-ALL vs. PMBL)
Median age, years (range)	13.0 (4–64)	11.0 (4–54)	13.5 (4–34)	27.5 (23–44)	34, 64	52		
M:F	32:20	23:8	6:6	1:5	2:0	0:1	0.129	**0.014**
**Clinical symptom**								
Cough	30/52 (57.7)	22/31 (71.0)	4/12 (33.3)	2/6 (33.3)	2/2 (100)	0/1 (0)	**0.055**	0.157
Dyspnea	12/52 (23.1)	11/31 (35.5)	1/12 (8.3)	0/6 (0)	0/2 (0)	0/1 (0)	0.161	0.151
Chest pain	15/52 (28.8)	9/31 (29.0)	1/12 (8.3)	4/6 (66.7)	0/2 (0)	1/1 (100)	0.299	0.157
Fever	16/52 (30.8)	10/31 (32.3)	4/12 (33.3)	0/6 (0)	1/2 (50.0)	1/1 (100)	1.000	0.162
**Clinical stage**								
Stage I	2/52 (3.8)	0/31 (0)	0/12 (0)	2/6 (33.3)	0/2 (0)	0/1 (0)		**0.023**
Stage II	13/52 (25.0)	1/31 (3.2)	7/12 (58.3)	3/6 (50.0)	1/2 (50.0)	1/1 (100)	**0.001**	**0.010**
Stage III	13/52 (25.0)	7/31 (22.6)	5/12 (41.7)	1/6 (16.7)	0/2 (0)	0/1 (0)	0.211	1.000
Stage IV	24/52 (46.2)	23/31 (74.2)	0/12 (0)	0/6 (0)	1/2 (50.0)	0/1 (0)	**0.000**	**0.001**
Tumor size (cm)	9.37 ± 3.24	10.31 ± 2.74	7.82 ± 4.77	6.60 ± 1.42			**0.266**	**0.015**
Ratio of TD to MTD	0.47 ± 0.17	0.52 ± 0.15	0.45 ± 0.24	0.28 ± 0.07			**0.579**	**0.004**
**Tumor compression or encasement**								
Blood vessels	29/35 (82.9)	23/23 (100)	3/6 (50.0)	2/4 (50.0)	1/2 (50.0)		**0.005**	**0.017**
Pericardium	23/35 (65.7)	18/23 (78.3)	3/6 (50.0)	1/4 (25.0)	1/2 (50.0)		0.305	0.065
Trachea	19/35 (54.3)	14/23 (60.9)	2/6 33.3)	1/4 (25.0)	2/2 (100)		0.364	0.294
**Complication**								
Pleural effusion	23/35 (65.7)	21/23 (91.3)	1/6 (16.7)	0/4 (0)	1/2 (50.0)		**0.001**	**0.001**
Pericardial effusion	14/35 (40.0)	13/23 (56.5)	1/6 (16.7)	0/4 (0)	0/2 (0)		0.169	0.098
Pneumonia	17/35 (48.6)	14/23 (60.9)	1/6 (16.7)	0/4 (0)	2/2 (100)		0.080	**0.041**

#### Histological subtypes and age groups

The 52 patients, ranging from 4 to 64 years (median 13 years), were classified as 31 pediatric patients (from 4 to 18 years, median 8 years) and 21 adult patients (from 20 to 64 years, median 28 years). The pediatric patients presented with T-LBL/T-ALL and CHL in 24 and 7 cases, respectively. The adult patients presented with T-LBL/T-ALL, PMBL, CHL, DLBCL, and MALT lymphoma in 7, 6, 5, 2, and 1 cases, respectively. Pediatric patients had a significantly higher prevalence of T-LBL/T-ALL (24/31 and 77.4% vs. 7/21 and 33.3%; *P* = 0.001) and lower incidence of PMBL (0/31 and 0% vs. 6/21 and 28.6%; *P* = 0.003) than adult patients.

The ages of patients with T-LBL/T-ALL, CHL, and PMBL ranged from 4 to 54 years (median, 11 years), 4 to 34 years (median, 13.5 years), and 23 to 44 years (median, 27.5 years), respectively. The two DLBCL patients were 34 and 64 years old, and the MALT patient was 52 years old. T-LBL/T-ALL and CHL occurred in both pediatric and young adult patients, whereas PMBL exclusively occurred in young adults.

#### Gender tendencies and age groups

Pediatric patients were more likely to be male predilection with a M:F ratio of 22:9, as compared with adults of 10:11 (*P* = 0.089). Adult patients with T-LBL/T-ALL showed a male predominance with a M:F ratio of 6:1, compared to pediatric patients of 17:7. The tendency of male predominance in T-LBL/T-ALL patients seemed to be increased with age. Patients with PMBL and CHL presented with female predominance with the M:F ratios of 1:5 and 6:6 respectively. Patients with T-LBL/T-ALL showed a higher male predominance than patients with PMBL (*P* = 0.014).

#### Initial presentations and age groups

Five patients had been identified during physical examination without symptoms. Forty-seven patients exhibited various symptoms, including cough (30/52, 57.7%), chest pain (15/52, 28.8%), fever (16/52, 30.8%), dyspnea (12/52, 23.1%), and neck enlargement (6/52, 11.5%). Cough was the most common symptom in T-LBL/T-ALL patients (22/31, 71.0%) and chest pain was often presented in PMBL patients (4/6, 66.7%). Pediatric patients showed higher frequency of dyspnea than adults (12/31 and 38.7% vs. 0/21 and 0%; *P* = 0.001).

#### Clinical stages and age groups

Stages I, II, III, and IV were present in 0 (0%), 4 (12.9%), 8 (25.8%), and 19 (61.3%) pediatric patients, respectively, as well as in 2 (9.5%), 9 (42.9%), 5 (23.8%), and 5 (23.8%) adult patients, respectively. Compared to adults, pediatric patients more frequently presented stage IV disease (19/31 and 61.3% vs. 5/21 and 23.8%; *P* = 0.008) and less frequently presented II disease (4/31 and 12.9% vs. 9/21 and 42.9%; *P* = 0.034). T-LBL/T-ALL patients presented higher frequency with stage IV disease than CHL (*P* = 0.000) and PMBL (*P* = 0.001) patients, and less frequency with stage II presentation than CHL (*P* = 0.001) and PMBL (*P* = 0.010) patients.

#### Clinical follow-up

Six pediatric patients with T-LBL/T-ALL died due to progression of the disease during the process of therapy, 22 patients were still under treatment, and the other 24 patients were followed up from 10 to 35 months (mean, 23.5 months).

### Radiological assessments

Radiological data of 50 cases were available, including computed tomography (CT), fluorodeoxyglucose positron emission tomography-computed tomography (PET-CT), and magnetic resonance imaging (MRI) in 50, 21, and 1 cases, respectively. Radiological data of 35 cases were available at initial diagnosis, and the radiological features are summarized in Table 4.

**Table 4:** Radiological characteristics of 52 cases with lymphomas involving the mediastinum.

*Case no.	Histological subtype	Location	Size (cm)	TD (cm)/MTD (cm) (ratio)	Compression or encasement			Complication		
					Blood vessels	Pericardiaum	Trachea	Pleural effusion	Pericardial effusion	Pneumonia
**Pediatric patients**										
1	T-LBL/T-ALL	Anterior M	11.3	11.3/17.2 (0.657)	Y	Y	Y	Y	Y	N
2	T-LBL/T-ALL	Anterior M	8.8	8.8/17.9 (0.492)	Y	Y	Y	Y	Y	Y
3	T-LBL/T-ALL	Anterior-superior M	11.6	11.6/16.3 (0.712)	Y	Y	Y	Y	Y	Y
4	T-LBL/T-ALL	Anterior and middle M	14.1	14.1/16.5 (0.856)	Y	Y	Y	Y	N	Y
5	T-LBL/T-ALL	Anterior M	13.5	13.5/18.5 (0.730)	Y	Y	N	Y	Y	Y
6	T-LBL/T-ALL	Anterior M	8.6	8.6/17.7 (0.486)	Y	Y	Y	Y	Y	N
7	T-LBL/T-ALL	Anterior-superior M	8.0	8.0/16.9 (0.473)	Y	N	Y	Y	Y	N
8	T-LBL/T-ALL	NA	NA	NA	NA	NA	NA	NA	NA	NA
9	T-LBL/T-ALL	NA	NA	NA	NA	NA	NA	NA	NA	NA
10	T-LBL/T-ALL	NA	NA	NA	NA	NA	NA	NA	NA	NA
11	T-LBL/T-ALL	Anterior M	6.0	NA	Y	N	N	N	N	N
12	T-LBL/T-ALL	NA	NA	NA	NA	NA	NA	NA	NA	NA
13	T-LBL/T-ALL	Anterior-superior M	6.0	6.0/18.9 (0.317)	Y	Y	Y	Y	N	Y
14	T-LBL/T-ALL	Anterior and middle M	12.0	12.0/19.4 (0.619)	Y	Y	Y	Y	Y	Y
15	T-LBL/T-ALL	Anterior-superior M	8.9	8.9/23.4 (0.380)	Y	Y	N	Y	N	Y
16	T-LBL/T-ALL	Anterior M	9.8	9.8/21.8 (0.450)	Y	Y	N	Y	Y	N
17	T-LBL/T-ALL	Anterior M	13.5	13.5/20.8 (0.649)	Y	Y	Y	Y	N	N
18	T-LBL/T-ALL	Anterior-superior M	5.7	5.7/15.1 (0.377)	Y	N	N	Y	N	Y
19	T-LBL/T-ALL	Anterior M	9.3	9.3/23.6 (0.394)	Y	Y	N	Y	Y	Y
20	T-LBL/T-ALL	Anterior-superior M	6.1	6.1/23.8 (0.256)	Y	N	Y	N	N	N
21	T-LBL/T-ALL	Superior M	10.7	10.7/22.2 (0.482)	Y	N	N	Y	N	N
22	T-LBL/T-ALL	Anterior and middle M	12.7	12.7/21.1 (0.602)	Y	Y	Y	Y	Y	Y
23	T-LBL/T-ALL	NA	NA	NA	NA	NA	NA	NA	NA	NA
24	T-LBL/T-ALL	Anterior M	12.0	12.0/24.6 (0.488)	Y	Y	N	Y	N	N
25	CHL	NA	NA	NA	NA	NA	NA	NA	NA	NA
26	CHL	Anterior M	4.0	4.0/16.7 (0.239)	N	N	N	N	N	N
27	CHL	Anterior-superior M	10.0	10.0/14.6 (0.685)	Y	Y	Y	N	N	N
28	CHL	NA	NA	NA	NA	NA	NA	NA	NA	NA
29	CHL	NA	NA	NA	NA	NA	NA	NA	NA	NA
30	CHL	Anterior-superior M	13.4	13.4/20.1 (0.658)	Y	Y	Y	Y	Y	Y
31	CHL	Anterior-superior M	3.0	NA	N	N	N	N	N	N
**Adult patients**										
32	T-LBL/T-ALL	Anterior M	12.0	12.0/24.9 (0.482)	Y	Y	Y	Y	Y	Y
33	T-LBL/T-ALL	NA	NA	NA	NA	NA	NA	NA	NA	NA
34	T-LBL/T-ALL	Anterior M	11.9	11.9/23.4 (0.508)	Y	Y	N	Y	N	Y
35	T-LBL/T-ALL	Anterior-superior M	9.8	9.8/22.9 (0.428)	Y	Y	Y	Y	Y	Y
36	T-LBL/T-ALL	Anterior M	14.8	14.8/24.2 (0.612)	Y	Y	Y	Y	Y	Y
37	T-LBL/T-ALL	NA	NA	NA	NA	NA	NA	NA	NA	NA
38	T-LBL/T-ALL	NA	NA	NA	NA	NA	NA	NA	NA	NA
39	CHL	Anterior-superior M	3.8	3.8/24.3 (0.165)	N	N	N	N	N	N
40	CHL	NA	NA	NA	NA	NA	NA	NA	NA	NA
41	CHL	Anterior M	12.7	12.7/24.3 (0.523)	Y	Y	N	N	N	N
42	CHL	NA	NA	NA	NA	NA	NA	NA	NA	NA
43	CHL	NA	NA	NA	NA	NA	NA	NA	NA	NA
44	PMBL	Anterior M	4.5	4.5/24.2 (0.186)	N	N	N	N	N	N
45	PMBL	NA	NA	NA	NA	NA	NA	NA	NA	NA
46	PMBL	NA	NA	NA	NA	NA	NA	NA	NA	NA
47	PMBL	Anterior M	7.4	7.4/22.1 (0.335)	Y	Y	N	N	N	N
48	PMBL	Anterior-superior M	7.0	7.0/26.9 (0.260)	N	N	N	N	N	N
49	PMBL	Anterior M	7.5	7.5/21.8 (0.344)	Y	N	Y	N	N	N
50	MALT	NA	NA	NA	NA	NA	NA	NA	NA	NA
51	DLBCL	Anterior M	10.6	10.6/25.2 (0.421)	Y	Y	Y	N	N	Y
52	DLBCL	Middle M	6.8	6.8/25.6 (0.266)	N	N	Y	Y	N	Y

Abbreviations: M, mediastinum; NA, not available; N, no; Y, yes.

^*^Case numbers are identical with the patient number used in Table 1.

The anterior or both the anterior and the superior mediastinum was the most common location of mediastinal lymphomas (31/35, 88.6%), followed by both the anterior and middle mediastinum (3/35, 8.6%), and the middle mediastinum (1/35, 2.8%).

The tumor diameters (TD) were variable, ranging from 3 to 14.8 cm (mean, 9.4 cm), with pediatric patient tumor masses ranging from 3 to 14.1 cm (mean, 9.5 cm) and adult patient tumor masses ranging from 3.8 to 14.8 cm (mean, 9.1 cm). Bulky masses were more frequently revealed in T-LBL/T-ALL (mean, 10.3 cm) than in CHL (mean, 7.8 cm) and PMBL (mean, 6.6 cm) cases. The ratios of TD to maximum transthoracic diameter (MTD) in patients with T-LBL/T-ALL were higher than those of PMBL patients (0.52 ± 0.15 vs. 0.28 ± 0.07; *P* = 0.004). The ratios of TD to MTD in pediatric patients were significantly higher than those of adult patients (0.52 ± 0.17 vs. 0.38 ± 0.14; *P* = 0.015).

**Figure 1:**
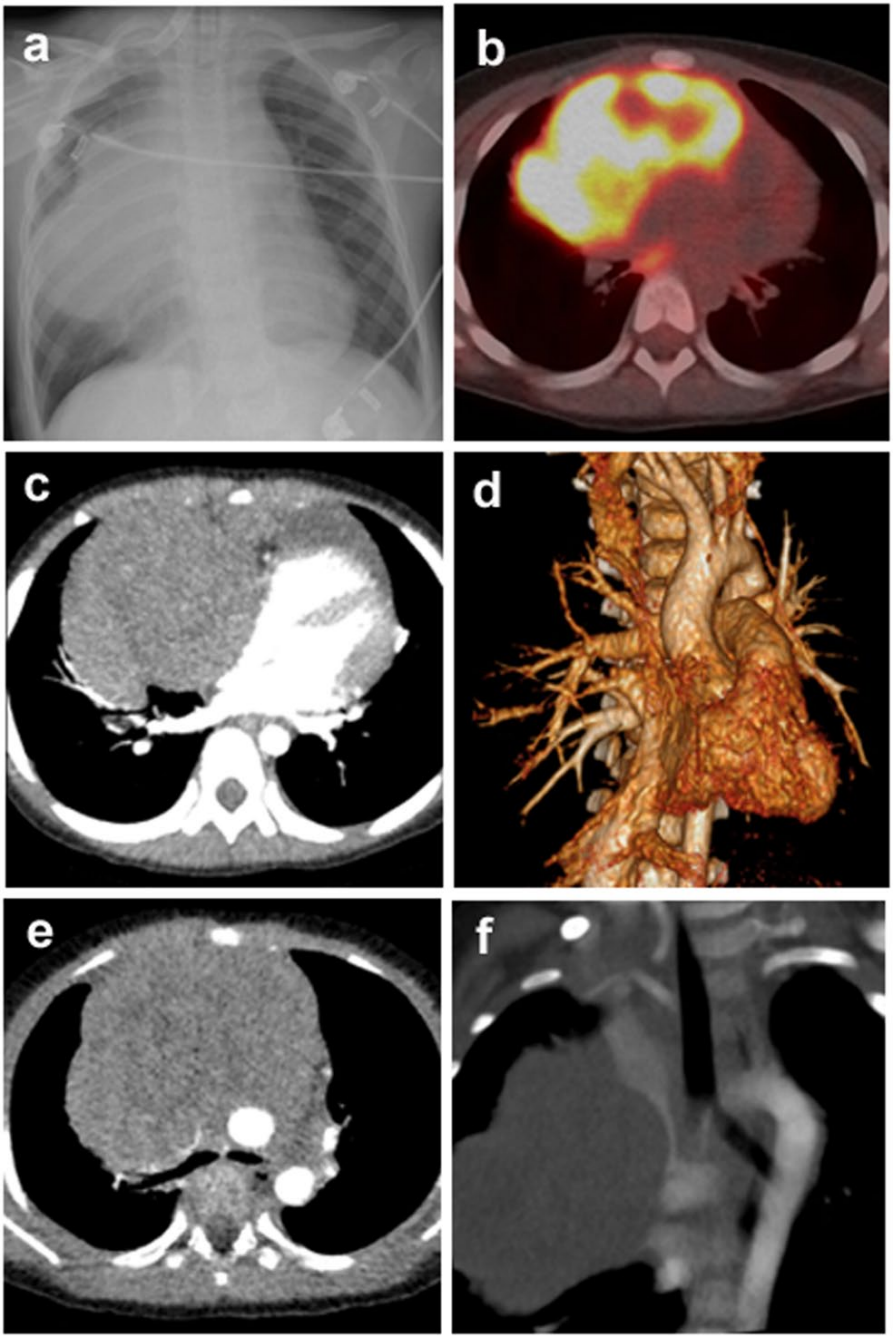
Radiological characteristics of T-LBL/T-ALL involving the mediastinum, case 1. (**a**) Chest X-ray image shows a large mass in the mediastinum. (**b**) PET-CT image shows increased FDG activity in the mediastinum. (**c**) Axial contrast-enhanced CT scan shows the compression of pericardiaum. (**d**) Three-dimensional volume rendered image of contrast-enhanced CT exhibits the compression of pericardiaum. (**e**) Axial contrast-enhanced CT scan shows vascular compression and tracheal encasement of the mass. (**f**) Maximal intensity projection of contrast-enhanced CT reveals the encasement of superior vena cava.

As shown in Fig. 1 and Table 4, tumor masses variably compressed or encased the blood vessels, pericardium, and trachea in 29 (29/35, 82.9%), 23 (23/35, 65.7%), and 19 (19/35, 54.3%) cases, respectively. T-LBL/T-ALL patients images exhibited greater tendency for compression of the blood vessels than CHL (*P* = 0.005) and PMBL (*P* = 0.017) patient.

Complications of lymphomas involving the mediastinum commonly included pleural effusion (23/35, 65.7%), pericardium effusion (14/35, 40.0%), and pneumonia (17/35, 48.6%). T-LBL/T-ALL patients presented with a higher frequency of pleural effusions than CHL and PMBL patients, (*P* = 0.001) for both, and pneumonia than PMBL patients (*P* = 0.041). Pediatric patients were likely higher prevalence of pleural effusion than adult patients (18/23 and 78.3% vs. 5/12 and 41.7%; *P* = 0.059).

## Discussion

Lymphomas involving the mediastinum occur in a wide age range and demonstrate various clinical symptoms and radiological findings. However, the clinicopathological and radiological differences between pediatric and adult patients have not been clearly defined in previous reports^1, 10^.

As reported previously, each histological subtype exhibited distinct clinicopathological features^3–6, 14^. However, comprehensive clinicopathological comparison among these subtypes is lacking in previous studies. In this study, pediatric patients exclusively presented with T-LBL/T-ALL and CHL, in contrast to adult patients, who presented with complex subtypes of T-LBL/T-ALL, CHL, PMBL, DLBCL, and MALT lymphomas. We further investigated the comparisons among different histological subtypes.

T-LBL/T-ALL is a highly aggressive neoplasm of lymphoblasts of T-cell origin. Due to the rapid growth of tumor cells, it is common for T-LBL/T-ALL patients to present with an advanced stage and unfavorable outcome as reported previously^15, 16^. In our results, T-LBL/T-ALL patients showed more frequent stage IV presentation than CHL and PMBL patients. Imaging of T-LBL/T-ALL patients commonly showed bulky masses, blood vessels compression, and pleural effusion.

PMBL is a common histological subtype in adult patients. PMBL patients are reported to have favorable clinical outcomes compared to patients with systemic DLBCL involving the mediastinum^17, 18^. In our study, PMBL occurred in young adults, who presented with a higher frequency of localized clinical stage than patients with T-LBL/T-ALL.

CHL occurs in both pediatric and adult patients^4^. As shown in our study, the most common histological subtype was NSCHL. Previous reports have shown that NSCHL generally presents with localized disease and a better prognosis than other subtypes of CHL^19^. Some patients have morphological features overlapping between NSCHL and PMBL, such as mediastinal gray-zone lymphoma, which makes pathological diagnoses challenging^20^. Our results showed that an immunohistochemical profile of positivity for CD15 and CD30 was helpful for the diagnosis of CHL. In addition, distinguishing PMBL from CHL could also be based on the value of CD23, which has been confirmed previously^21^.

Thymic MALT lymphomas are quite rare and occur most frequently in Asian females from 40 to 60 years of age. They often show an indolent course and localized disease^22^. Thymic MALT lymphomas are distinct from MALT lymphomas of other sites in several ways, including gene abnormalities and geographic distribution^23^. In the current study, the MALT lymphoma patient was a 52-year-old female who presented with stage II. In our study, 2 cases of systemic DLBCL with secondary mediastinal involvement were identified. Despite the pathological similarities to PMBL, DLBCL exhibited more aggressive clinical features and a poor outcome.

Although the discrimination of histological subtypes of lymphomas may be the most important reason for the clinical and radiological features, age-related differences of tumor biology, host characteristics, or treatment protocols also contribute to different outcomes between pediatric and adult patients. For example, pediatric type follicular lymphoma is considered to be a separate entity which differs from usual adult follicular lymphoma in clinical and pathological features^24, 25^.

Age is an important factor for lymphoma. Non-Hodgkin lymphoma (NHL) in pediatric patients present biological and epidemiological peculiarities that if better understood could help optimize their outcome. In order to investigate the influence of age on biology of lymphomas, we have compared pediatric and adult lymphomas in the same anatomic sties. As we reported recently, B-cell lymphomas involving the Waldeyer’s ring had distinctive clinicopathological characteristics in pediatric patients compared to adult counterparts. A subset of cases belonged to the new entity of IRF4/MUM1 positive lymphoma^26, 27^.

Of note, for appropriate management of pediatric and adult lymphomas, different staging systems have been used. The original Ann Arbor staging system^28^ and the updated Lugano classification^29^ were designed without input from the pediatric NHL disease entities. The Murphy Classification^30^ and the revised Pediatric Non-Hodgkin Lymphoma Staging system (IPNHLSS)^31^ facilitated more precise staging for children and adolescents with NHL. However, few studies compared pediatric and adult patients with mediastinal NHL by the different staging systems. We assessed the clinical stages of pediatric and adult T-LBL/T-ALL patients using the revised IPNHLSS and the Lugano classification, respectively.

Despite of a population over 1300 million in China, to date, English information on mediastinal lymphomas of Chinese populations is limited. We described 52 cases of lymphomas involving the mediastinum, which exhibited a wide age range, heterogeneous histological subtypes, various clinical presentations and complex radiological manifestation. Compared to adult lymphomas, pediatric lymphomas presented distinctive histological subtypes, clinical behaviors, and radiological features. The limitation of this study is the relatively small number of cases, and further studies are needed for more detailed understanding.

Accordingly, this is the first multidisciplinary English report comprehensively compared the differences between Chinese pediatric and adult patients with mediastinal lymphomas and may provide new insight into the understanding of mediastinal lymphoma using different staging systems.

## Materials and Methods

### Case selection

Cases were obtained from a single Chinese institution: Shanghai Xinhua Hospital. A total of 506 patients with the mediastinal mass were reviewed between November 2011 and December 2016. Among these cases, 52 cases of lymphomas involving the mediastinum were identified and included in this study. The pathological diagnoses were originally established according to the criteria of the 2008 World Health Organization classification. All experimental protocols were approved by the Ethics Committee of Xinhua Hospital Affiliated to Shanghai Jiaotong University School of Medicine and performed in accordance with the approved guidelines of the institution. The appropriate informed consent was obtained from all subjects under the institutional review board-approved protocol.

### Age grouping, clinical information, and staging

The patients were arbitrarily defined as 2 groups, including pediatric group under 19 years of age and adult group over 19 years of age. The clinical data, including the age, gender, date of initial diagnosis, symptoms, clinical stage, serum lactate dehydrogenase level, medical records, and surgical records, were reviewed. The disease stages of CHL and adult NHL patients were assessed according to the Lugano Classification^29^ and pediatric NHL patients to the IPNHLSS^31^.

### Radiological modalities

The patients were identified by imaging assessments including chest X-ray, CT, MRI, or PET-CT.

### Histological and immunohistochemistry analyses

Formalin-fixed paraffin-embedded tissues were stained with hematoxylin and eosin at the initial diagnosis. Immunohistochemistry was performed using the following panel of monoclonal and polyclonal antibodies: CD20 (clone L26, DAKO, Glostrup, Denmark); CD79a (clone 1.10E + 04, Leica Biosystems, Wetzlar, Germany); PAX5 (clone R1, DAKO); CD10 (clone 56C6, Leica Biosystems); BCL6 (clone P1F6, DAKO); MUM1 (clone MUM1p, DAKO); Ki-67 (clone MIB-1, DAKO); Terminal deoxynucleotidyl transferase (TDT, clone SP150, DAKO); CD2 (clone AB75, DAKO); CD3 (clone LN10, Leica Biosystems); CD4 (clone SP35, DAKO); CD7 (DAKO); CD8 (clone SP16, DAKO); CD1a (clone SP157, DAKO); CD34 (clone QBEnd/10, DAKO); CD43 (clone DF-T1, DAKO); CD99 (clone HO36-1.1, DAKO); CD117 (clone C-KIT, DAKO); CD45 (clone PAN-LCAL, DAKO); CD33 (clone WM-54, DAKO); MPO (clone SP72, DAKO); CD15 (clone C3D-1, DAKO); CD30 (clone Ber-H2, DAKO); CD23 (clone SP23, DAKO); and Epithelial membrane antigen (EMA, clone GP1.4, Leica Biosystems).

### Statistical analysis

Statistical analyses were performed with SPSS version 17.0 software (SPSS Inc., Chicago, IL, USA). Chi-squared and Fisher’s exact tests were used to determine correlation in the frequencies between groups. The TD to MTD were summarized using the mean and standard deviation. Two tailed independent samples t-test were used to assess statistical significance in comparisons between the pediatric and adult patients.

## Electronic supplementary material

Supplementary Tables

## References

[1] Takeda S (2003). Clinical spectrum of primary mediastinal tumors: a comparison of adult and pediatric populations at a single Japanese institution. J Surg Oncol..

[2] Temes R (2000). Primary Mediastinal Malignancies in Children: Report of 22 Patients and Comparison to 197 Adults. Oncologist..

[3] Cortelazzo S, Ponzoni M, Ferreri AJ, Hoelzer D (2011). Lymphoblastic lymphoma. Crit Rev Oncol Hematol..

[4] Mauch PM (1993). Patterns of presentation of Hodgkin disease. Implications for etiology and pathogenesis. Cancer..

[5] Steidl C, Gascoyne RD (2011). The molecular pathogenesis of primary mediastinal large B-cell lymphoma. Blood..

[6] Weissferdt A, Moran CA (2011). Primary MALT-type lymphoma of the thymus: a clinicopathological and immunohistochemical study of six cases. Lung..

[7] Wilson WH (2014). A prospective study of mediastinal gray-zone lymphoma. Blood..

[8] Duwe BV, Sterman DH, Musani AI (2005). Tumors of the mediastinum. Chest..

[9] Liu T (2017). Mediastinal lesions across the age spectrum: a clinicopathological comparison between pediatric and adult patients. Oncotarget.

[10] Savage KJ (2003). The molecular signature of mediastinal large B-cell lymphoma differs from that of other diffuse large B-cell lymphomas and shares features with classical Hodgkin lymphoma. Blood..

[11] Allan BJ (2013). An analysis of 73 cases of pediatric malignant tumors of the thymus. J Surg Res..

[12] Gun F (2012). Mediastinal masses in children: experience with 120 cases. Pediatr Hematol Oncol..

[13] Patel JL (2012). The immunophenotype of T-lymphoblastic lymphoma in children and adolescents: a Children’s Oncology Group report. Br J Haematol..

[14] Vitolo U (2016). Extranodal diffuse large B-cell lymphoma (DLBCL) and primary mediastinal B-cell lymphoma: ESMO Clinical Practice Guidelines for diagnosis, treatment and follow-up. Ann Oncol..

[15] Le Gouill S (2003). Adult lymphoblastic lymphoma: a retrospective analysis of 92 patients under 61 years included in the LNH87/93 trials. Leukemia..

[16] Coustan-Smith E (2009). Early T-cell precursor leukaemia: a subtype of very high-risk acute lymphoblastic leukaemia. Lancet Oncol..

[17] Abou-Elella AA (1999). Primary mediastinal large B-cell lymphoma: a clinicopathologic study of 43 patients from the Nebraska Lymphoma Study Group. J Clin Oncol..

[18] Rosenwald A (2003). Molecular diagnosis of primary mediastinal B cell lymphoma identifies a clinically favorable subgroup of diffuse large B cell lymphoma related to Hodgkin lymphoma. J Exp Med..

[19] Allemani, C., Sant, M., De Angelis, R., Marcos-Gragera, R. & Coebergh, J. W. EUROCARE Working Group. Hodgkin disease survival in Europe and the U.S.: prognostic significance of morphologic groups. *Cancer*. **107**, 352–360 (2006). 10.1002/cncr.21995 16770772

[20] Gualco G, Natkunam Y, Bacchi CE (2012). The spectrum of B-cell lymphoma, unclassifiable, with features intermediate between diffuse large B-cell lymphoma and classical Hodgkin lymphoma: a description of 10 cases. Mod Pathol..

[21] Salama ME, Rajan Mariappan M, Inamdar K, Tripp SR, Perkins SL (2010). The value of CD23 expression as an additional marker in distinguishing mediastinal (thymic) large B-cell lymphoma from Hodgkin lymphoma. Int J Surg Pathol..

[22] Lorsbach RB, Pinkus GS, Shahsafaei A, Dorfman DM (2000). Primary marginal zone lymphoma of the thymus. Am J Clin Pathol..

[23] Inagaki H (2002). Primary thymic extranodal marginal-zone B-cell lymphoma of mucosa-associated lymphoid tissue type exhibits distinctive clinicopathological and molecular features. Am J Pathol..

[24] Liu Q (2013). Follicular lymphomas in children and young adults: a comparison of the pediatric variant with usual follicular lymphoma. Am J Surg Pathol..

[25] Swerdlow SH (2016). The 2016 revision of the World Health Organization classification of lymphoid neoplasms. Blood..

[26] Chen L, Al-Kzayer LF, Liu Y, Liu T (2017). B-cell lymphomas involving Waldeyer’s ring characterized by distinctive clinical and histopathological features: a comparison of pediatric to adult patients. Oncotarget.

[27] Chen L (2017). IFR4/MUM1-positive lymphoma in Waldeyer ring with co-expression of CD5 and CD10. Pediatr Blood Cancer.

[28] Lister TA (1989). Report of a committee convened to discuss the evaluation and staging of patients with Hodgkin’s disease: Cotswolds meeting. J Clin Oncol..

[29] Cheson BD (2014). Recommendations for initial evaluation, staging, and response assessment of Hodgkin and non-Hodgkin lymphoma: The Lugano classification. J Clin Oncol.

[30] Murphy SB (1980). Classification, staging and end results of treatment of childhood non-Hodgkin’s lymphomas: dissimilarities from lymphomas in adults. Semin Oncol..

[31] Rosolen A (2015). Revised International Pediatric Non-Hodgkin Lymphoma Staging System. J Clin Oncol.

