# Supplementary material for: Comparison of pediatric and adult lymphomas involving the mediastinum characterized by distinctive clinicopathological and radiological features
Source: Sci Rep. 2017 May 31;7:2577. doi: 10.1038/s41598-017-02720-1 (PMC5451435; doi:10.1038/s41598-017-02720-1)
Supplement: Supplementary file 1 — Supplementary Tables [file 41598_2017_2720_MOESM1_ESM.pdf]

Comparison of pediatric and adult lymphomas involving the mediastinum characterized by distinctive clinicopathological and radiological features

Lei Chen[1], Mingsong Wang[2], Hua Fan[3], Fengqing Hu[2], Tingting Liu[4]

[1]Department of Pathology, Xinhua Hospital, Shanghai Jiaotong University School of Medicine, Shanghai, China;

[2]Department of Thoracic Cardiovascular Surgery, Xinhua Hospital, Shanghai Jiaotong University School of Medicine, Shanghai, China;

[3]Department of Radiology, Xinhua Hospital, Shanghai Jiaotong University School of Medicine, Shanghai, China;

[4]Department of Pediatric Hematology/Oncology, Xinhua Hospital, Shanghai Jiaotong University School of Medicine, Shanghai, China

Correspondence:

Tingting Liu, Department of Pediatric Hematology/Oncology, Xinhua Hospital, Shanghai Jiaotong University School of Medicine, Kongjiang Road 1665, Shanghai, 200092, China.

E-mail: liuting0552390@yahoo.co.jp

Short title: Lymphomas involving the mediastinum

Supplementary Table 1. Immunohistochemical features of 31 cases with T-LBL

*Case no.	CD3	CD2	CD5	CD7	CD4	CD8	CD43	TDT	CD1a	CD34	CD99	CD117	LCA	CD10	Ki-67	BCL2
1	+	+	+	+	+	-	+	-	+	-	N	-	+	+	90%+	-
2	+	-	+	+	+	-	N	N	-	-	N	N	N		N	N
3	+	N	+	+	N	N	N	N	-	-	+	-	-	N	90%+	N
4	+	+	-	+	+	-	+	+	+	-	+	-	F+	+	95%+	+
5	+	N	+	+	+	+	+	F+	+	-	+	-	N	-	90%+	-
6	+	+	+	+	F+	+	+	F+	+	-	+	-	+	F+	60%+	+
7	+	N	N	+	N	N	N	+	N	+	+	N	+	+	90%+	N
8	+	+	+	+	-	-	N	N	-	-	N	N	N	N	N	N
9	+	N	+	+	N	N	N	+	+	-	-	N	+	N	75%+	N
10	+	N	N	N	N	N	+	F+	N	-	N	N	N	-	90%+	-
11	+	+	+	+	N	N	N	N	+	+	N	N	N	N	N	N
12	+	+	N	N	N	N	N	+	N	-	N	N	N	+	90%+	+
13	+	+	+	+	+	-		+	N	+	N	-		-		
14	+	N	-	+	+	+	+	+	+	-	+	-	+	+	90%+	+
15	+	N	+	+	+	+	+	+	+	-	-	-	N	+	70%+	+
16	+	+	+	+	-	+	-	+	+	-	+	N	+	-	80%+	N
17	+	-	+	+	+	-	N	+	-	-	+	-	+	+	70%+	N
18	-	-	N	-	-	-	+	Part+	-	Part+	-	+	+	-	80%+	N
19	+	+	-	+	N	N	N	+	N	-	N	N	N	+	90%+	+
20	-	-	-	+	-	-	+	-	-	+	-	Part+	+	-	40%+	+
21	+	N	N	N	N	N	N	+	N	N	N	N	N	+	90%+	+
22	+	N	-	+	N	N	N	+	+	-	+	N	N	-	-	+
23	+	N	+	+	+	+	+	+	+	+	+	-	N	+	80%+	F+

**24**	+	**N**	**N**	-	**N**	**N**	**N**	-	+	-	+	**N**	+	+	**60%+**	-
**32**	+	**N**	-	+	**N**	**N**	+	+	**N**	**N**	**N**	**N**	-	+	**40%+**	**N**
**33**	+	-	**N**	+	**N**	**N**	+	+	**N**	-	+		-	-	**90%+**	
**34**	+	+	+	+	**N**	**N**	**N**	**N**	-	**N**	**N**	**N**	**N**	**N**	**N**	**N**
**35**	+	-	+	+	-	-	-	-	-	-	+	-	+	**N**	**N**	**N**
**36**	+	+	+	+	-	-	+	-	+	-	-	-	+	+	**90%+**	+
**37**	+	+	-	+	-	+	+	+	**N**	-	**N**	**N**	**N**	-	**90%+**	+
**38**	+	**N**	-	**N**	**N**	**N**	**N**	+	**N**	**N**	+	**N**	+	-	**60%+**	**N**

Abbreviations: F, focal; N, not done; +, positive; -, negative.

*Case numbers are identical with the patient number used in Table 1.

Supplementary Table 2. Immunohistochemical features of 21 cases with B-cell lymphomas

*Case no.	Histological subtype	CD20	PAX5	CD79a	CD43	CD3	CD7	CD10	BCL6	MUM1	Ki-67	P53	BCL2	EMA	CD15	CD30	CD23
25	CHL	-	+	N	N	-	-	-	N	N	N	N	N	N	-	+	N
26	CHL	-	+	-	N	-	-	-	N	-	30%+	N	N	N	+	+	N
27	CHL	F+	+	-	N	N	+	-	F+	N	60%+	+	-	-	-	+	N
28	CHL	-	F+	-	+	+	-	-	-	+	40%+	N	-	N	-	+	+
29	CHL	+	-	+	N	-	-	-	-	-	10%+	N	-	-	+	+	-
30	CHL	F+	+	N	N	F+	N	N	N	+	N	N	N	N	+	+	N
31	CHL	-	F+	-	-	-	-	-	-	+	30%+	-	-	-	+	+	-
39	CHL	-	F+	N	N	-	N	N	N	N	30%+	N	N	N	+	+	+
40	CHL	-	+	-	+	+	+	-	-	+	10%+	+	-	-	+	+	+
41	CHL	-	-	-	+	+	N	-	-	+	60%+	-	-	-	+	+	-
42	CHL	-	+	-	+	+	-	-	N	N	30%+	N	-	-	+	+	+
43	CHL	+	+	+	N	N	N	N	N	N	20%+	N	N	N	+	+	+
44	PMBL	+	+	+	-	-	-	-	-	N	50%+	N	-	+	-	F +	-
45	PMBL	+	N	N	N	N	N	-	N	N	60%+	N	N	N	N	N	+
46	PMBL	+	+	+	N	-	-	-	+	+	30%+	N	+	-	-	-	+
47	PMBL	+	+	+	+	F+	F+	-	+	+	40%+	N	+	-	-	F+	+
48	PMBL	-	+	-	N	-	N	-	-	+	50%+	-	+	-	-	+	N
49	PMBL	+	+	+	-	-	-	-	+	-	70%+	N	+	-	-	-	+
50	MALT lymphoma	+	+	+	N	-	N	-	N	F+	10%+	-	+	-	N	N	-
51	DLBCL	+	N	+	N	-	N	-	+	+	70%+	N	+	N	N	-	N
52	DLBCL	+	+	+	N	-	-	N	N	N	50%+	N	N	N	N	N	N

Abbreviations: F, focal; N, not done; +, positive; -, negative.

*Case numbers are identical with the patient number used in Table 1.
